# Aptamer-Functionalized Nano-Biosensors

**DOI:** 10.3390/s91210356

**Published:** 2009-12-21

**Authors:** Tai-Chia Chiu, Chih-Ching Huang

**Affiliations:** 1 Department of Applied Science, National Taitung University, 684, Section 1, Chunghua Road, Taitung, 95002, Taiwan; 2 Institute of Bioscience and Biotechnology, National Taiwan Ocean University, 2 Beining Road, Keelung, 20224, Taiwan

**Keywords:** nanomaterials, aptamers, biosensors, metal ions, proteins, cells

## Abstract

Nanomaterials have become one of the most interesting sensing materials because of their unique size- and shape-dependent optical properties, high surface energy and surface-to-volume ratio, and tunable surface properties. Aptamers are oligonucleotides that can bind their target ligands with high affinity. The use of nanomaterials that are bioconjugated with aptamers for selective and sensitive detection of analytes such as small molecules, metal ions, proteins, and cells has been demonstrated. This review focuses on recent progress in the development of biosensors by integrating functional aptamers with different types of nanomaterials, including quantum dots, magnetic nanoparticles (NPs), metallic NPs, and carbon nanotubes. Colorimetry, fluorescence, electrochemistry, surface plasmon resonance, surface-enhanced Raman scattering, and magnetic resonance imaging are common detection modes for a broad range of analytes with high sensitivity and selectivity when using aptamer bioconjugated nanomaterials (Apt-NMs). We highlight the important roles that the size and concentration of nanomaterials, the secondary structure and density of aptamers, and the multivalent interactions play in determining the specificity and sensitivity of the nanosensors towards analytes. Advantages and disadvantages of the Apt-NMs for bioapplications are focused.

## 1. Introduction

Aptamers (Apt) that are short single-stranded (ss) nucleic acids (DNA or RNA) have been used to bind from small solutes to peptide to proteins to cells, viruses, or parasites, with high affinity [1-6]. These functional nucleic acids can fold into well-defined three-dimensional structures to form binding pockets and clefts for the specific recognition and tight binding of any given molecular target. They can be produced synthetically and commonly identified *in vitro* from vast combinatorial libraries that comprise trillions of different sequences by a process known as systematic evolution of ligands by exponential enrichments (SELEX) [7-10], that has recently been fully automated. Automation has reduced *in vitro* aptamer selection times from months to days. Typically, after 5 to 15 cycles of the SELEX process, the library is reduced to contain only a small number of aptamers which exhibit particularly high affinity to a target. The equilibrium dissociation constants (*K*_d_) of aptamers to targets are usually in the range of picomolar (pM) to micromolar (μM), similar to those of antibodies for antigens [9,10]. Having such high affinity, aptamer-based homogeneous and heterogeneous sensor systems have been employed for the detection of metal ions, small organic molecules, proteins, and nucleic acids. Fluorescence, colorimetry, and electrochemistry are common detection modes used in these sensor systems [11-16].

The past few years have witnessed progressive advance in the synthesis and characterization of a variety of nanomaterials (NMs), including metallic nanoparticles (NPs), quantum dots (QDs), magnetic NPs, silica (SiO_2_) NPs, carbon nanotubes (CNTs), and so on [17]. Having large surface area, and unique size, shape and composition-dependent physical and chemical properties including surface plasmon resonance (SPR), surface enhanced Raman scattering (SERS), fluorescence, electrochemistry, magnetism, and/or catalytic activity, those NMs are promising candidates as basic building and signaling elements for fabrication of biosensors with great sensitivity [18-21].

In the past few years, integration of functional aptamers into NMs has become a new interdisciplinary field that aims at providing new hybrid sensing systems (sensors) for specific and sensitive molecular recognition [15,16,21]. This novel integration has yielded various types of sensors for selective and sensitive detection of a wide range of analytes such as adenosine, cocaine, mercuric ion, and thrombin. Sensors are devices that respond to physical or chemical stimuli and produce detectable signals [22-24]. A sensor requires at least two steps: target recognition and signal transduction [21-24]. The target recognition element can be any chemical or biological entity such as small organic molecules, peptides, proteins, nucleic acids, carbohydrates, or even whole cells. In these studies aptamers were used as the target recognition elements. Ideally, this element should have high affinity (low detection limit), high specificity (low interference), wide dynamic range, fast response time, and long shelf life. Signal transduction elements are responsible for converting molecular recognition events into physically detectable signals such as fluorescence, color, electrochemical signals, and magnetic resonance image changes. Metallic NPs, magnetic NPs, QDs, and CNTs are the signal transduction elements in these studies.

Among the developed aptamer-based nanosensors, metallic NPs such as gold nanoparticles (Au NPs) and silver nanoparticles (Ag NPs) are the most common. Metallic NPs that possess strongly distance-dependent optical properties and large surface areas have emerged as important colorimetric materials [17]. Because Au NPs possess many chemical and physical properties of interest, they have been most commonly used for the fabrication of miniaturized optical devices, sensors, and photonic circuits, as well as in medical diagnostics and therapeutics. One of their most important properties is a strong SPR absorption with extremely high extinction coefficients (10^8^∼10^10^ M^–1^ cm^–1^) in the visible wavelength range. The extinction cross-sections of the particles and the wavelengths at which they absorb and scatter light both depend on their size and shape, the dielectric properties (refractive index) of the surrounding medium, and their interactions with neighboring particles. The SPR band undergoes red shifts upon increasing the size of the Au NPs. Unlike spherical Au NPs, Au nanorods and Au-Ag nanorods have two SPR bands; for example, a transverse band at 508–532 nm and a longitudinal band at 634–743 nm for Au-Ag nanorods, depending on their aspect ratio (length/width) [25,26]. The SPR frequency of Au NPs changes dramatically upon varying the refractive index of the local environment and/or the average distance between Au NPs. Systems based on analyte-induced aggregation of Au NPs have been employed for the colorimetric detection of cells, nucleic acids, proteins, small molecules, and metal ions [27-31]. Commonly, these sensing systems are based on analyte-induced crosslinking of Au NPs, which causes color changes as a result of electronic dipole-dipole coupling between neighboring particles and scattering. Dispersed Au NPs having interparticle distances substantially greater than their average particle diameter appear red, whereas the color of the aggregates changes to purple as the interparticle distance drops below the average particle diameter.

Recently, DNA-functionalized NMs have been used in a variety of detection modes for proteins, oilgonucleotides, metal ions, and other small molecules [14-16,21,22]. In this review, we will discuss recent advances in the preparation, characterization, and applications of NMs, including QDs, magnetic NPs, Au NPs, and CNTs, that are conjugated with aptamers. We highlight advantages and disadvantages of functionalized NMs through various detection modes, including colorimetry, fluorescence, electrochemistry, SPR, and, mass spectrometry for the detection of small molecules and proteins. The functionalized NMs are selective and sensitive for the analytes, showing their great potential in biosensing and bioimaging.

## 2. Aptamer Nanosensors for Small Molecules

Relative to biopolymers, small molecules have far fewer moieties for aptamer binding. Thus aptamers that recognize small molecules of interest are relatively rare. Most representative aptamers and their targeted small analytes are listed in Table 1. When the aptamers bind to the target, they usually change their structures from random and coiled conformation to G-quartet or other structures. Apt-Au NPs are one of the most common Apt-NMs for detecting of various analytes. An example is to detect cysteine down to 100 nM using a oligonucleotide-functionalized Au NP probe based on the selective coordination of T-T mismatch with mercury ion (Hg^2+^) [48]. When cysteine bound the purple aggregates linked by oligonucleotide with Hg^2+^ complexed T-T mismatches, the Hg^2+^ is sequestered from the aggregate through cysteine complexation, thereby lowering the *T*_m_ at which DNA duplexes dissociated and the corresponding purple-to-red color change took place.

### 2.1. Adenine Nucleotides and Their Derivatives

#### 2.1.1. Colorimetry

Adenine nucleotides play critical roles in the regulation and integration of cellular metabolism and biochemical pathways in cell physiology [61]. During muscle contraction, adenosine triphosphate (ATP) is hydrolyzed enzymatically to adenosine monophosphate (AMP) or to adenosine diphosphate (ADP) prior to furnish phosphoric acid and energy during metabolism. ATP is generated in the muscle by further enzymatic action. The ubiquitous involvement of adenosine nucleotides in the metabolism, active transport, and mechanical work of myocardial cells, makes their accurate measurement essential for investigating the biochemical, structural, and functional manifestations of cardiac ischemia. ATP has also been used as an indicator for cell viability and cell injury [62]. Therefore, determination of ATP is essential in biochemical study as well as clinical diagnosis.

**Figure 1.**
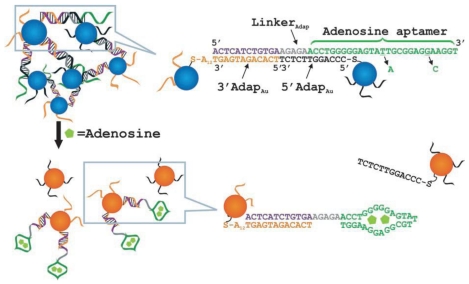
Schematic representation of colorimetric detection of adenosine. The DNA sequences are shown in the right side of the figure. The A_12_ in 3′Adap_Au_ denotes a 12-mer polyadenine chain. In a control experiment, a mutated linker with the two mutations shown by the two short black arrows was used. Reprinted with permission from Reference [32].

Liu *et al.* developed a colorimetric aptamer-based nanosensor for adenosine as shown in Figure 1 [32]. The sensor contained a linker DNA (Linker_Adap_) molecule that could be divided to three segments according to its function: (1) the first segment (in purple) hybridized with a Au NP functionalized with 3′-thiol-modified DNA (3′Adap_Au_); (2) the second segment (in gray) hybridized with the last five nucleotides of a 5′-thiol-modified DNA on another Au NP (5′Adap_Au_); and (3) the third segment (in green), the aptamer sequence for adenosine, hybridized with the other seven nucleotides on the 5′Adap_Au_. 3′Adap_Au_ and 5′Adap_Au_ were assembled with Linker_Adap_ to form aggregates, which displayed a faint purple color when suspended in solution. In the presence of adenosine, the aptamer changed its structure to bind adenosine. As a result, only five base pairs were left to hybridize with 5′Adap_Au_, which was unstable at room temperature. Therefore, the 5′Adap_Au_ dissociated from the 3′Adap_Au_, resulting in disassembly of the aggregates. Upon disassembly, the color of the system changed from purple to red. Quantitative analysis was performed by monitoring the absorbance ratio (A_522_/A_700_) at one minute after the addition of adenosine, with a concentration range from 0.3 to 2.0 mM. This similar strategy using a different DNA linker and aptamers could also be applied to the detection of cocaine with the range from 50 to 500 μM. Liu *et al.* also used the same adenosine aptamer to build a model system to develop an aptamer-based lateral flow device [33]. The adenosine and cocaine aptamer-linked Au NP aggregates were immobilized onto a lateral flow device separately. In the presence of target molecules, the NPs would be disassembled owing to binding of target molecules by the aptamers. The dispersed NPs then migrated along the membrane and were captured to form a red line. The system provided LODs of ca. 20 and 10 μM for adenosine and cocaine, respectively. Taking advantage of the optical properties of both CdSe/ZnS QDs and Au NPs, Liu *et al.* further demonstrated a method for the detection of adenosine and cocaine in one pot [38]. QDs were used to encode aptamer-linked nanostructures sensitive to adenosine and cocaine separately. The nanostructures also contained Au NPs that served as quenchers. Addition of target analytes disassembled the nanostructures and resulted in the increased emission from QDs. The LODs for adenosine and cocaine were 50 and 120 μM, respectively.

A sensing system for highly sensitive adenosine detection based on surface inhibition was developed by Wang *et al.* [36]. The aptamer was first immobilized on SPR gold film with its ssDNA structure. The aptamer possessing random and coiled ssDNA structure could be hybridized with Au NPs tagged complementary ssDNA and resulted in a large change in SPR signal. However, after adenosine was added to the SPR cell, the aptamer changed its structure from ssDNA to tertiary structure that could not be hybridized with Au NPs-tagged complementary ssDNA. Thus, the change of SPR signal resulted in the hybridization reaction between aptamer and Au NPs-tagged complementary ssDNA decreased upon increasing the number of tertiary-structured aptamers, which is linearly proportional to the concentration of adenosine over the range 1 nM to 1 μM.

Aptamer-based colorimetric sensors are usually prepared from aptamers and Au NPs through covalent bonding. Thiol derivated aptamers are most commonly used to prepare functionalized Au NPs through strong Au-S bonding. Thiol derivated aptamers are more expensive relative to unmodified aptamers (without thiol modification). In addition, the purity of thiol derivated aptamers is sometimes problematic. The DNA after conjugation on the NP surface may change its conformation and thus alter its binding affinity towards the target. As a result, reduced sensitivity and specificity may occur. To prevent these disadvantages, a universal aptamer-target binding readout strategy using unmodified Au NPs was developed [43]. By using both anti-ATP aptamer and i-motif as the models, this method allowed selective detection of ATP even by the naked eye. Equivalent anti-ATP aptamer and its complementary oligonucleotides were first hybridized to form the duplex. Then the duplex system was incubated with a certain amount of ATP for 30 min at 37 °C to induce the structural switching. Interestingly, upon adding Au NPs to the duplex solution followed by addition of appropriate amounts of salts, the solution almost instantaneously (within seconds) changed its color from red to blue. In contrast, if there was only ssDNA in the solution, the color remained red. By using this method, ATP could be detected as low as ∼ 2 μM by the naked eye. The LOD by absorption detection was reduced to 0.6 μM.

Chen *et al.* used Apt-Au NPs to demonstrate a colorimetric sensing approach for the determination of ATP [44]. In the absence of ATP, the color of the Apt-Au NPs solution changed from wine-red to purple as a result of salt-induced aggregation. Binding of the ATP to the Apt-Au NPs induced folding of the aptamers on the Au NP surfaces into G-quartet and/or an increase in charge density. As a result, the Apt-Au NPs solution was wine-red in color in the presence of the ATP under high salt conditions. The LOD for ATP detection was 10.0 nM.

#### 2.1.2. Fluorescence

Fluorescent NMs including QDs, Au nanodots, and Ag nanocrystals have become interesting materials for sensing and imaging of analytes of interest [63-66]. QDs provide advantages over traditional organic fluorophores, including photostability, chemical stability, and narrower emission but broader excitation spectra [67]. In addition, the QD surface can be modified easily with chemicals or biopolymers. Chen *et al.* developed a new fluorescent method for the detection of ATP using CdSe/ZnS QDs [46]. This approach used three different oligonucleotides: 3′-biotin-modified DNA that bound to streptavidin conjugated 605-nm emissive QD (605QD) through biotin-streptavidin interaction; 3′-Cy5-labelled DNA; and a capture DNA consisting of an ATP aptamer and a sequence which could hybridize with the two aforementioned DNA sequences. In the absence of ATP, the capture DNA hybridized with both 3′-biotinmodified DNA and 3′-Cy5-labelled DNA, which induced close proximity between 605QD and Cy5. Under such a condition, 605QD was excited at 488 nm, and Cy5 emitted fluorescence at 670 nm in virtue of fluorescence resonance energy transfer (FRET). In contrast, when aptamer interacted with ATP, its conformation changed, leading to the dissociation of 3′-Cy5-labelled DNA from the hybridization complex. As a result, the fluorescence intensity changed. The LOD of this approach for ATP was 2.4 μM.

Song *et al.* reported a novel AMP biosensor based on the aptamer-induced disassembly of fluorescent and magnetic nano-silica sandwich complexes with a direct detection of 0.1 μM [47]. The sensor was composed of three components: magnetic silica microspheres (MSMPs) functionalized with 30-amino-modified DNA (MSMP probe), fluorescent silica nanoparticles (FNPs) functionalized with 50-amino-modified DNA (FNP probe), and a linker DNA (detection probe). In the absence of AMP, FNPs were separated together with MSMPs away from the aqueous medium by magnetic separation, leading to weak fluorescence in the aqueous medium. However, with the addition of AMP, the dissociated FNPs remained in the aqueous medium that exhibits strong fluorescence. The LOD for AMP was as low as 0.1 μM.

#### 2.1.3. Mass Spectrometry

**Figure 2.**
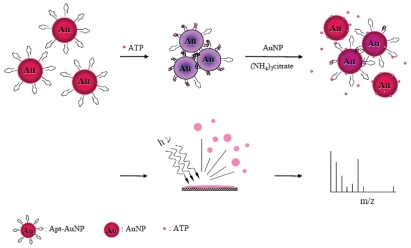
Illustration of the interactions of ATP with Apt-Au NPs and Au NPs. Reprinted with permission from Reference [45].

Recently, NPs have become interesting materials for mass spectrometry, mainly because they can act as probe to recognize targeted molecules and as matrices like the organic molecules used in matrix assisted laser desorption mass spectrometry (MALDI-MS) to assist desorption/ionization of the analytes. Most common NPs used in mass spectrometry are Au NPs, TiO_2_ NPs, and Fe_3_O_4_ NPs [68]. Using aptamer-modified 13-nm Au NPs as selective probes and unmodified Au NPs as the surface-assisted laser desorption/ionization (SALDI) matrices for the determination of ATP by mass spectrometry was demonstrated by Huang *et al.* [45]. Figure 2 describes the strategy of the two-step sample preparation for analysis of ATP by SALDI mass spectrometry (SALDI-MS) using Apt-Au NPs are effective LDI matrices. A thiol-modified ATP-binding aptamer that has a specific affinity (*K*_d_∼6 μM) with ATP was introduced to Au NPs. The ATP-binding aptamer was covalently attached to the surfaces of Au NPs through Au-S bonding. By using Apt-Au NPs as selective probes and Au NPs as LDI matrices, this approach provided the LOD for ATP at a signal-to-noise ratio of 3 of 0.48 μM. Because Au NPs and Apt-Au NPs provided high selectivity toward glutathione and ATP, respectively, they were also used for the analysis of the two analytes in lysates of human red blood cells after simple sample pretreatment. The estimated concentration of ATP and glutathione in the cell lysate were 1.9 ± 0.3 and 0.94 ± 0.06 mM (*n* = 3), respectively.

### 2.2. Cocaine

Cocaine is a natural stimulant derived from the leaves of the *Erythroxylum coca* plant, found mainly in South America. For illicit use, cocaine is often taken in its hydrochloride form through nasal insufflations or intravenous injection, or as its free base through smoking. Because of its potent stimulant effect on the central nervous system, cocaine is a personal health safety risk that can result in serious societal problems. There are more than two million people in the US alone are frequent cocaine users [69].

**Figure 3.**
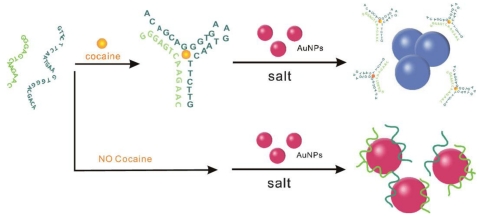
Scheme for the engineered cocaine aptamer and the visual detection of cocaine based on the red-to-blue color change of Au NPs. Reprinted with permission from Reference [39].

A novel bioassay strategy was designed by Zhang *et al.* to detect cocaine using 13-nm Au NPs and engineered DNA aptamers [39]. The aptamer was engineered to possess two pieces of random, coil-like ssDNA, which reassembled into the intact aptamer tertiary structure in the presence of cocaine. Au NPs effectively differentiated between these two states *via* their characteristic SPR based color changes as shown in Figure 3. By comparing the ratio for A_520_/A_650_, cocaine was selectively detected within minutes with a LOD at 2 μM.

Self-assembly of labeled aptamer CdSe/ZnS QDs in the presence of cocaine stimulated a FRET process [40], allowing detection of cocaine down to 1.0 μM. The functionalized QDs consisted of two sub-units of aptamers, in which one was linked to QDs, and the other was subjected to self-assembly in the presence of cocaine through forming cocaine-aptamer complexes. A similar nanosensor using CdSe/ZnS QDs (605 QD) that was conjugated with aptamers to recognize cocaine was developed to detect cocaine down to 0.5 μM [41]. Cocaine induced the conformation change in the aptamer, leading to occurrence of FRET between 605 QD and Cy 5 and Iowa Black RQ.

Shlyahovsky *et al.* introduced an autonomous aptamer-based machine that amplified the recognition events between the aptamer and its substrate [70]. The LOD of cocaine for this fluorescence approach was 5.0 μM. Freeman et al. reported that two different enzymes tethered to the anti-cocaine aptamer fragments, or nicotinamide adenine dinuclotide/enzyme tethered to the anti-cocaine aptamer fragments, self-assembled, in the presence of cocaine, to supramolecular structures that activate biocatalytic cascades [71]. Because the mutual orientation of the two enzymes, or the cofactor-enzyme units, on the aptamer fragments was regulated by the formation of the aptamer-substrate supramolecular structure, control over the functional reactivity of the biocatalystic system was dictated by the concentration of the substrate. This method enabled the analysis of cocaine with a LOD of 0.5 μM.

## 3. Aptamer Nanosensors for Metal Ions

In this section, we focus on aptamer nanosensors for the detection of several metal ions, including Pb^2+^, Hg^2+^, and K^+^. The monitoring of toxic metal ions (e.g., Pb^2+^ and Hg^2+^) in aquatic ecosystems is an important issue because these contaminants can have severe effects on human health and the environment [72]. For example, lead can cause renal malfunction and inhibit brain development [73] and mercury can damage brain, heart, and kidneys [74]. Potassium ions are involved in many biological functions, including nerve transmission, regulation of blood pressure and pH, enzyme activation, and the formation of collagen or elastin. Abnormal levels of K^+^ ions in biological fluids can cause muscle cramps or weakness, nausea, diarrhoea, frequent urination, dehydration, paralysis, and changes in heart rhythms [75].

### 3.1. Lead Ions (Pb^2+^)

**Figure 4.**
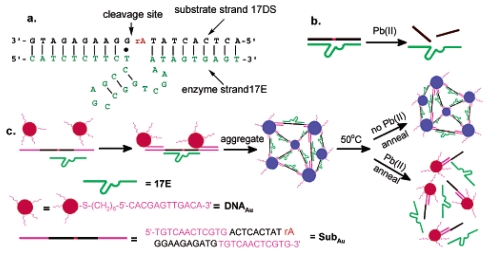
(a) Secondary structure of the “8-17” DNAzyme system that consists of an enzyme strand (17E) and a substrate strand (17DS). The cleavage site is indicated by a black arrow. Except for a ribonucleoside adenosine at the cleavage site (rA), all other nucleosides are deoxyribonucleosides. (b) Cleavage of 17DS by 17E in the presence of Pb^2+^. (c) Schematics of DNAzyme-directed assembly of Au NPs and their application as biosensors for metal ions such as Pb^2+^. In this system, the 17DS has been extended on both the 3′ and 5′ ends for 12 bases, which are complementary to the 12-mer DNA attached to the 13-nm gold nanoparticles (DNA_Au_). Reprinted with permission from Reference [57].

Liu *et al.* constructed a series of colorimetric lead biosensors using DNAzyme-directed assembly of Au NPs [57-59]. As shown in Figure 4, the system consists of 5′-thiol-modified 12-mer DNA attached to 13-nm-diameter Au NPs, a DNAzyme (17E), and its substrate (Sub_Au_). The sequence of the Sub_Au_ was designed so that it could hybridize specifically to a DNA-Au on each end, while maintaining the color. However, in the presence of Pb^2+^ ions, the 17E catalyzed hydrolytic cleavage of Sub_Au_ and prevented the formation of NP aggregates. A red color appeared as a result. The LOD of this sensor system was down to sub-micromolor level for Pb^2+^ ions. Similar approaches were further demonstrated for the detection of Pb^2+^ ions within 10 min [58,59]. In this study, the authors conducted a detailed study of the sensing system, which identified that “tail-to-tail” NPs alignment and large nanoparticle size (42 nm diameter) were the major determining factors for a fast color change. Elbaz *et al.* used DNAzyme cascades for amplified sensing of Pb^2+^ ions [76]. DNAzyme cascades activated by Pb^2+^-dependent DNAzymes yielded the horseradish peroxidase-mimicking catalytic nucleic acids that enabled the colorimetric detection of Pb^2+^ with a LOD corresponding to 10 nM.

### 3.2. Mercuric Ions (Hg^2+^)

**Figure 5.**
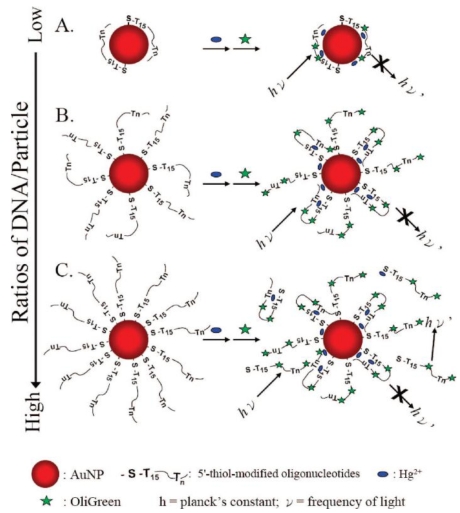
Schematic representation of Hg^2+^ nanosensors at various DNA-to-Au NP molar ratios: (a) <30, (b) 30–50, and (c) ≥60. Reprinted with permission from Ref. [51].

Liu *et al.* demonstrated a Apt-Au NPs probe for sensing Hg^2+^ using the formation of DNA-Hg^2+^ complexes through T-Hg^2+^-T coordination to control the negative change density of the DNA strands adsorbed onto Au NPs [53]. Upon formation of Hg^2+^-DNA complexes, the conformation of the poly-T*_n_*-ssDNA changed to the folded structures. As a result of the decreased zeta potential on each Au NPs and the reduced degree of electrostatic repulsion among Au NPs, aggregation of the Au NPs occurred and the color of the solution changed from red to purple that was detectable by the naked eye. By plotting the Ex_650/520_ absorption ratio of Au NPs against Hg^2+^ concentration, the LOD was obtained to be 250 nM. A new highly selective and sensitive technique for the detection of Hg^2+^ using DNA- functionalized Au NPs and OliGreen (OG) was demonstrated by the same group [51]. Figure 5 shows the sensing mechanism, which is based on the release of DNA molecules from the Au NP surface to the bulk solution and their subsequent specific interactions with OliGreen. When Hg^2+^ ions interact with the thymidine units of the DNA molecules, the conformations of these DNA derivatives change from linear to hairpin structures, causing the release of some of the DNA molecules from the surface of the Au NPs into the bulk solution. OG molecules then interact with these free DNA species, resulting in an increase in the fluorescence at 525 nm upon excitation at 480 nm. The DNA-OG complexes fluoresce about 1,000-fold more intensely than does the free OG, which is only weakly fluorescent. Hg^2+^ could be detected at concentrations as low as 25 nM. Another simple and sensitive aptamer-based colorimetric detection of Hg^2+^ using unmodified Au NPs as probe was shown by Li *et al.*, with a five orders of magnitude of 100 μM to 1 nM [31]. The simple and cost-effective approach provided an LOD of 0.6 nM for Hg^2+^.

**Figure 6.**
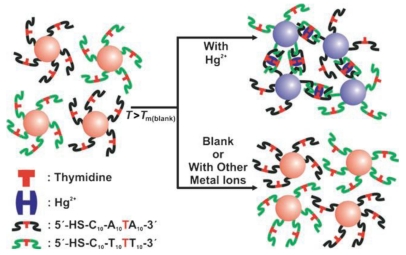
Colorimetric detection of Hg^2+^ using DNA-Au NPs. Reprinted with permission from Ref. [49].

A colorimetric method to detect Hg^2+^ using Apt-Au NPs in aqueous solution with very high selectivity and sensitivity was demonstrated by Lee *et al.* [49]. Hg^2+^ selectively bound to the T-T sites led to the formation of aggregates from mismatched strands and raised the *T*_m_ of the resulting structures. As shown in Figure 6, adding an aliquot of an aqueous solution of Hg^2+^ at a designated concentration to a solution of the Apt-Au NPs aggregates formed at room temperature. The *T*_m_ of the hybridized Apt-Au NPs proportionally correlated with the concentration of Hg^2+^, leading to up to a 10 °C increase in *T*_m_ for the aggregates. Without Hg^2+^, the aggregates melt with a dramatic purple-to-red color changed at about 46 °C. In the presence of Hg^2+^, however, the aggregates melted at temperatures higher than 46 °C. This assay provided an LOD of 100 nM and opened up the possibility of point-of-use applications. A chip-based scanometric method for the detection of Hg^2+^ was developed by the same group [50]. This assay utilized a variant of the approach to create a high-sensitivity, chip-based assay for Hg^2+^ by using probes capable of hybridizing with surface immobilized oligonucleotides to form duplexes with T-T mismatches. Hg^2+^ binding to these sites would create more stable duplex structures and raised the temperature associated with dehydration. The LOD of this approach was determined to be 10 nM.

### 3.3. Potassium Ions (K^+^)

Wang *et al.* demonstrated a colorimetric probe for K^+^ by switching the structure of DNA aptamer using unmodified Au NPs [56]. This G-rich aptamer is a random-coil ssDNA in solution. Upon binding to K^+^, the aptamer folded to a four-stranded tetraplex structure (G-quartet) *via* intermolecular hydrogen bonding between guanines [77,78]. Addition of salt screened electrostatic repulsion between negatively charged Au NPs and resulted in aggregation of Au NPs that led to color changes from red to purple [56].

## 4. Aptamer Nanosensors for Proteins and Cells

Aptamers have comparable affinities for target analytes, and offer a number of competitive advantages over antibodies [79]. Aptamers have markedly lower molecular weights than antibodies (usually below 20,000), secondary structures are easily predictable, lower immunogenicity, and binding affinity can rival antibodies. Aptamers are relatively stable under a wide range of buffer conditions, high temperature and resistant to physical or chemical degradation. In addition, aptamers are not prone to the irreversible denaturation that often alters the biologically activity of antibodies. Moreover, they can be synthesized efficiently and reliably by using established phosphoramidite chemistry, whereas antibody preparation often requires animals or cell cultures. Aptamers are also amenable to a wide variety of chemical modifications, such as radioscopic or fluorescent reporters, affinity tags for molecular recognition, 2′(deoxy)ribose ring modifications, such as 2′F- and 2′*O*-methyl, or construction from unnatural L-nucleotides to make aptamers nuclease resistant [80]. Thus far, many aptamers have been identified, and some of them are very close to becoming marketable drugs [81].

Nevertheless, engineering these aptamers for enhanced performance or new functions remains virtually at initial stage. One of the most significant advantages of aptamers in molecular assembly for multivalent binding is that they can be used to prepare polyvalent ligand-protected NPs easily, for example, with 5′- or 3′-amino or thiol groups [82]. Integration of aptamer with NPs provides new hybrid systems that combine the specific molecular recognition or catalytic properties of functional aptamers with the diverse and strong signal transduction of NPs. This capability presents great potential in making Apt-NPs with greatly enhanced functions, such as ultra high and tunable binding affinity, multiplex detection, and high resistance against nuclease digestion, that are important for developing new materials for biosensing of proteins [21,83]. For example, Hernandez *et al.* presented an optical aptamer sensor based on single Au NPs plasmon resonances (sometimes also called localized surface plasmon resonance) for detecting avidin [84] and Maehashi *et al.* reported a label-free protein biosensors based on aptamer-modified single-walled CNT field effect transistor (SWCNT-FET) for the detection of IgE in the nM range [85]. In the following section, we will discuss recent advances in the detection of thrombin and platelet-derived growth factors (PDGFs) that have been mostly demonstrated using aptamers functionalized NMs.

### 4.1. Thrombin

Aptamers have been used for the detection of thrombin that is involved in the blood clotting process [86]. Thrombin is a coagulation protein that plays many roles in the coagulation cascade—it converts soluble fibrinogen into insoluble strands of fibrin—as well as catalyzing many other coagulation-related reactions [87]. Therefore, thrombin is usually considered as an important target when searching for anti-coagulants and antithrombotics to interfere in the blood coagulation [88]. Moreover, thrombin is considered as a useful tumor marker in the diagnosis of pulmonary metastasis [89]. The prominent structural features of human α-thrombin are the location of the catalytic triad within a deep canyon-like active site cleft and the presence of two extended surfaces that are mainly composed of positively charged residues and are referred to exosite 1 and exosite 2. Exosite 1 is required for thrombin binding to several thrombin substrates (fibrinogen, thrombin receptor, and heparin cofactor II) and ligands (thrombomodulin and glycoprotein 1b). Exosite 2 that is located close to the carboxy-terminal B chain helix is involved in heparin and prothrombin fragment 2 binding [86-89].

Disorders in blood clotting are tightly linked to many serious health issues, including heart attacks and strokes [90]. Therefore, thrombin is typically the target in anticoagulation therapy for these diseases. Two most well known thrombin binding aptamers are TBA_15_ that is 15 bases long (TBA_15_) and binds to exosite 1 and TBA_27_, that is 27 bases long and interacts with exosite 2 [91,92]. TBA_15_ with thrombin having a dissociation constant *K*_d_ around 100 nM is the first DNA thrombin binding aptamer was selected by Bock *et al.* [91]. Later, TBA_27_ with very high affinity aptamers (*K*_d_ = 0.5 nM) was elected by Tasset *et al.* [92]. As a potential anticoagulant, only TBA_15_ should have the enzymatic inhibitory functions required for thrombin-mediated coagulation because it interacts with the fibrinogen-binding exosite 1.

#### 4.1.1. Absorption

Unlike the analyte-induced crosslink of Au NPs, a label-free aptamer-based colorimetric sensing of thrombin using unmodified Au NP probes has been developed [93]. Unfolded ssDNA aptamer (TBA_27_) could bind to citrate-capped Au NPs through DNA base-gold electrostatic interactions. Thus, the unfolded ssDNA would adsorb onto the Au NPs and helped to enhance the Au NPs' stability against salt-induced aggregation. The folded ssDNA (e.g., G-quadruplex) possessing a relatively rigid structure prevented the exposure of the DNA bases to the Au NPs and the high density of negative charges increased the repulsion between the DNA and the Au NPs. Thus the G-quadruplex DNA structures could not adsorb on the Au NPs and lost the ability to protect the Au NPs. By carefully controlling salt concentration and the ratio of TBA_27_ to Au NPs ([TBA_27_]/[Au NPs]), this approach allowed detection of thrombin, with linear range from 0 to 167 nM and LOD of 0.83 nM.

The major disadvantage of the colorimetric sensors based on Au NPs for the detection of small molecules or proteins in solution is the interference from the color of background, resulting in a decrease in detection sensitivity of the sensors. Based on this idea, Wang *et al.* developed a dot-blot assay to detect thrombin by thrombin adsorbed nitrocellulose membrane and Apt-Au NPs conjugates [94]. The immobilized thrombin could bind to the Apt-Au NPs, and then using Au NPs for signal amplification that was based on their catalytic function of reducing Ag ions to grow NPs of identical composition or core-shell structures. A red color change representing the thrombin concentration after silver ions reduction could be read directly by eye. This device allowed detection of thrombin at the range of 0.115 to 9.25 pmole in 1% plasma. A similar strategy that using silica-gold core-shell NP as signal reporter was studied by Jana *et al.*, which allowed detection of thrombin at nanomolar concentrations by the naked eye [95].

Xu *et al.* reported Apt-Au NPs as probes in aptamer-based dry-reagent strip biosensor for thrombin analysis [96]. Since thrombin has two binding sites for aptamers, by attaching one aptamer to test zone of strip and another to Au NP, the presence of thrombin would link the Au NPs to the test zone surface. By recording the optical responses of the test zone with a portable strip reader display, the biosensor provided a linear response for thrombin over the concentration range of 5–100 nM, with a detection limit of 2.5 nM. The authors also demonstrated that aptamer-based dry-reagent strip biosensor were comparable to antibodies-based strip biosensor and successfully for detection of thrombin low as 0.6 pmol in human plasma samples.

#### 4.1.2. Fluorescence

In addition to playing a major role as color reporters in colorimetric sensing, Au NPs can also be excellent quenchers for organic dyes in their proximity, due to an increase in their nonradiative rate and a decrease in the dye's radiative rate [97]. By taking the advantage of Au NPs as an efficiency quencher, Wang *et al.* reported a thrombin biosensor mediated the fluorescence quenching between dye-labeled oligonucleotide and Apt-Au NPs [98]. Tetramethylrhodamine-labeled oligonucleotide (TAMRA-oligonucleotide) was hybridized with aptamer functionalized Au NPs. Upon recognition of the thrombin by the aptamers, the TAMRA-oligonucleotide was released and the fluorescence was recovered. This method allowed detection of thrombin at a concentration down to nM.

Choi *et al.* reported that the photoluminescence (PL) of TBA_15_ functionalized PbS QDs (3–6 nm) could be selectively quenched upon binding to thrombin *via* charge transfer from thrombin to the QDs [99]. The charge transfer occurred most likely in a way that an electron was transferred from a functional group in thrombin (e.g., amine) to the QD conduction band, and a hole moved in the opposite direction, resulting in a decrease of the absorption and PL intensities. This strategy provided a linear detection region from 0 to 30 nM of thrombin and yielded a detection limit of ∼1 nM.

**Figure 7.**
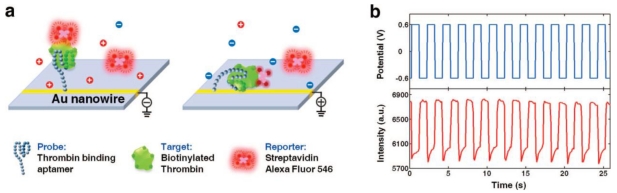
(a) A scheme showing an electrically modulated fluorescence protein assay including a thrombin-binding aptamer probe grafted on an Au nanowire, the target, a biotinylated thrombin, and the reporter, a fluorophore-labeled streptavidin. (b) Electrical potential applied on the nanowire (top) and modulated fluorescence measured from a sample at a 100 nM thrombin concentration (bottom) are shown synchronously versus time. Reprinted with permission from Ref. [100].

An electrically modulated fluorescence assay was demonstrated for thrombin through a single molecule assembled on an Au nanowire (Au NW) by manipulating the molecule with an electrical potential applied on the nanowire [100]. The scheme of probe-target-reporter sandwich assay is shown in Figure 7. Biotinylated thrombin was captured by aptamer assembled Au NW and then labeled by a fluorescent streptavidin reporter. By applying an alternating electrical potential on the Au NW, the probe-target-reporter complex was attracted toward or repelled from the Au NW, which modulated its fluorescence accordingly due to the surface energy transfer between the fluorescence reporter and NW. It was demonstrated that the molecular modality could be unequivocally correlated with the modulated fluorescence, which enabled the specific fluorescence from a single thrombin molecule to be unambiguously distinguished from background noise and nonspecific fluorescence. The LOD of the assay for thrombin in buffer solution was 100 fM, and the linear dynamic range of the assay could extend from 100 fM to 100 nM.

#### 4.1.3. Electrochemistry

Electrochemical aptamer biosensors based on NPs labels and a binding-induced label-free detection have been proven as one of the most powerful tools for protein analysis [101]. Most of bioelectrochemical aptamer sensor for determination of thrombin was based on the sandwich system of electrode-aptamer/thrombin/Apt-NPs as the sensing platform. These assays took advantage of the amplification potential of NPs carrying numerous tags or catalytic labels for ultra sensitive detection of proteins. Polsky *et al.* reported aptamer functionalized Pt NPs that acted as catalytic labels *via* reduction of H_2_O_2_ for the amplified electrochemical detection of thrombin [102]. The sensitivity of the method for detection of thrombin with an LOD of 1 nM.

He *et al.* developed a bioelectrochemical method for the detection of thrombin through directly detecting the redox activity of adenine nucleobases of aptamer using a pyrolytic graphite electrode [103]. Thrombin captured by immobilzed anti-thrombin antibody on microtiter plates, was detected by Apt-Au NPs bio bar codes. The adenine nucleobases were released by acid or nuclease from Au NPs bound on microtiter plates. Differential pulse voltammetry was employed to investigate the electrochemical behaviors of the purine nucleobases. Because the NP carried a large number of aptamers per thrombin binding event, there was substantial amplification and thus thrombin could be detected at a very low level of detection (0.1 ng/mL). This method was validated by the detection of thrombin in fetal calf serum with minimum background interference. For improvement of detection sensitivity for thrombin, a three-level cascaded impedimetric signal amplification was developed by Deng *et al.* [104]. Apt-Au NPs acted as the first-level signal enhancer, enlarged Apt-Au NPs were as the second-level signal amplification by nicotinamide adenine dinucleotide (NADH) and HAuCl_4_, and the redox probe [Fe(CN)_6_rsqb;^3-/4−^ was as the third-level signal amplification. Enlargement of Apt-Au NPs integrated with negatively charged surfactant (SDS) capping could not only improve the detection sensitivity of the impedimetric sensor but also presented a simple and general model for the signal amplification of the impedimetric sensor. This approach allowed the detection of thrombin at a concentration down to 100 fM.

In addition to tunable luminescence properties, QDs offer an electrodiverse population of electrical tags as needed for multiplexed bioanalysis [105]. Hansen *et al.* used QDs tracers (CdS and PbS) for designing multi-analyte electrochemical aptamer biosensors for thrombin and lysozyme [106]. This sensing platform involved in the co-immobilization of two thiolated aptamers, along with binding of the corresponding QD-tagged proteins (CdS QDs labeled thrombin and PbS QDs labeled lysozyme) on a gold surface, addition of the protein sample, and monitoring the displacement through stripping voltammetric detection of the remaining QDs. This method was sensitive and selective, allowing the simultaneous quantification of pM levels of the target proteins.

#### 4.1.4. Other Techniques

Other than as a colorimetric reporter or fluorescence quencher, Apt-Au NPs have been used in SERS for thrombin detection. Raman scattering cross-section of a molecule in SERS spectroscopy based on molecules residing at or near the surface of certain nanostructured metals can be increased by factors up to 10^14^–10^15^, that is comparable to fluorescence [107]. This great enhancement is presumably from the large electromagnetic (EM) field produced by hot spots, which reside in the nanoscale junctions or interstices in metal nanostructures such as dimers or aggregates. Moreover, SERS has the inherent advantages over fluorescence that narrower bandwidth and provide richer spectral information. Wang *et al.* developed a SERS sensor for thrombin recognition using Au NPs labeled with aptamer and Raman reporters (rhodamine 6G) [108]. A sensing interface with a sandwich type system of gold substrate-TBA/thrombin/TBA-Au NPs was fabricated, therefore, Au NPs would be captured on the gold substrate upon the addition of thrombin, resulting in an enhanced SERS signal. EM hot spots could be fabricated by deposition of Ag NPs on Au NPs and the large EM coupling effect was presumably produced at the hot spots between Au NPs and Ag NPs where the Raman reporters resided. Taking advantage of the sensitivity of SERS and the specificity of aptamer to thrombin recognition, this system allowed detection of thrombin as low as 0.5 nM. Recently, Cho *et al.* reported a sensing mechanism based on thrombin induced displacement of Raman probe (methylene blue) modified TBA_15_ from Au NPs [109]. The SERS signal decreased with increasing thrombin concentration over the range of 0.1 nM to 1 μM, with an LOD of 0.1 nM. In addition, the sensor allowed detection of 1 nM thrombin in the presence of 10% fetal calf serum.

Superparamagnetic iron oxide NPs (SPIOs) probes have emerged as a class of novel contrast and tracking agents for medical imaging [110,111]. When used as a contrast agent for magnetic resonance imaging (MRI), SPIOs allow researchers and clinicians to enhance the tissue contrast of an area of interest by increasing the relaxation rate of water. SPIOs are most often magnetite (Fe_2_O_3_/Fe_3_O_4_), and have crystal-containing regions of unpaired spins. These magnetic domains are disordered in the absence of a magnetic field, but when a field is applied, the magnetic domains align to create a magnetic moment much greater than the sum of the individual unpaired electrons [110,111]. The cross-linking of dextran-coated superparamagnetic iron oxide (CLIO) NPs functionalized with different biomolecules have been used for detection of different targets including oligonucleotides, proteins, enzymatic activities, viruses, and enantiomeric impurities [110-113]. It has been shown these functional CLIO NPs assemblies create a distinctive magnetic phenomenon called magnetic relaxation switching (MRS) and result in induced enhancement of spin-spin relaxation time of adjacent water protons [112]. Yigit *et al.* prepared aptamer conjugated CLIO NPs (Apt-CLIO NPs) for selective detection of thrombin down to 25 nM thrombin in 0.5-fold diluted human serum [113]. This sensing strategy was based on thrombin induced aggregated structure of Apt-CLIO NPs that led to decreases in the spin-spin relaxation time, resulted in decreased the brightness of MRI. The advantage of this Apt-CLIO NPs probe over other optical sensor for thrombin was that MRI signal is much less vulnerable to changes in background colors or fluorescence from biological media, such as serum.

Recently, one-dimensional nanostructures, such as CNT and Au NW, have been successfully demonstrated as sensitive biological sensors [114]. It has been reported that the real-time detection of single viruses, small molecules, and proteins is possible with biosensors using Au NW or CNT transistors as the active transducer [114]. In terms of FET technology, aptamers provide a preferable choice, because they are smaller in size than the Debye length. As a result, the binding event between the aptamers and the target proteins can occur within the electrical double layer in buffer solution, and therefore, changes in the charge distribution within proximity to the CNT can easily be detected by FET. Moreover, the density of the immobilized aptamers on the CNT channels can be controlled, and a high density of aptamers can easily be prepared. Aptamers modified SWCNT-FET biosensor for detecting thrombin was demonstrated [115]. The 5′-amine-modified aptamer was immobilized onto the side wall of a CNT transistor through covalent bonding with carbodiimidazole-activated Tween 20 pretreated CNT. Binding of thrombin to the aptamer induced a sharp drop in the conductance, while the addition of elastase as a control molecule did not affect conductance. A detection limit of 10 nM was reported in this work.

### 4.2. Platelet-Derived Growth Factors (PDGFs)

Platelet-derived growth factor (PDGF) is a growth factor protein found in human platelets; it has growth-promoting activity toward fibroblasts, smooth muscle cells, and glial cells [116]. The PDGF family of growth factors consists of five different disulfide-linked dimers: PDGF-AA, PDGF-AB, PDGF-BB, PDGF-CC, and PDGF-DD, which exert their biological effects through their receptors, PDGFR-α and PDGFR-β. Binding of the receptors to PDGF is known to activate intracellular tyrosine kinase, leading to autophosphorylation of the cytoplasmic domain of the receptor as well as phosphorylation of other intracellular substrates [116]. In particular, it plays a significant role in blood vessel formation (angiogenesis), the growth of blood vessels from already existing blood vessel tissue [117]. Uncontrolled angiogenesis is a characteristic of cancer [117]. Many tumor cell lines have since been shown to produce and secrete PDGFs, some of which also express the cognate PDGF receptors; the paracrine effect on the tumor stroma and, in some tumor cell lines, autocrine growth stimulation by PDGF are therefore possible [117]. In view of its importance, traditional antibody-based radioisotopic methods and ELISA techniques have been developed for the detection of PDGF [118]. Antibodies to PDGF are the most potent and specific antagonists of PDGF. The known inhibitors of PDGF include suramin, neomycin, and peptides derived from the PDGF amino acid sequence, but either they are too toxic or they lack sufficient specificity for practical applications [118-121]. To overcome these disadvantages, aptamer-based optical sensors have been used to determine PDGF [122-124]. Nonetheless, they suffer from low sensitivity and selectivity, which could hamper their effectiveness in detection of PDGF in biological samples. Recently, many Apt-NP-based sensors have been developed for highly sensitive detection of PDGFs in biological samples.

#### 4.2.1. Absorption

**Figure 8.**
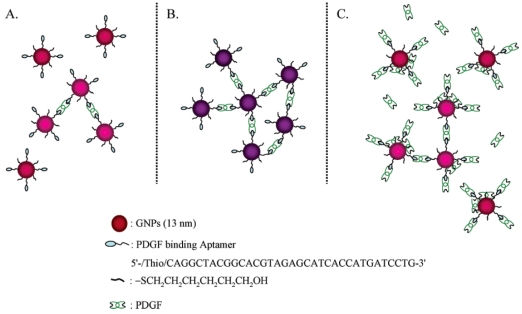
Schematic representation of the aggregation of Apt-Au NPs in the presence of PDGFs at (a) low, (b) medium, and (c) high concentrations. Reprinted with permission from Reference [126].

The aptamer selected for PDGF has a strong binding affinity toward PDGF (*K*_d_∼nM) [125]. The consensus secondary structure motif of the PDGF aptamer is a three-way helix junction with a conserved single-stranded loop at the branch point, in which the helix junction domain represents the core of the structural motif required for high-affinity binding. A highly specific sensing system using Apt-Au NPs was developed for the colorimetric detection of PDGF [126]. The color of Apt-Au NPs changed from red to purple at low concentration (<400 nM) as a result of aggregation with cross-linking between the Au NPs. Interestingly, the color was reversed to red at very high PDGF concentrations (>400 nM) due to the repulsion and steric effects because the surface of the Apt-Au NPs quickly becomes saturated with PDGF molecules through aptamer-PDGF binding (Figure 8). By plotting the ratios of the extinction coefficients of the Apt-Au NPs at 650 and 530 nm against the concentrations of PDGF-AA, the linear increased and decreased ranges of the extinction ratio were 25–75 and 75–200 nM, respectively. Furthermore, a homogeneous assay was developed to detect the PDGF receptor-*β* (PDGFR-*β*) at a concentration as low as 3.2 nM, on the basis of the competition between the Apt-Au NPs and PDGFR-*β* for PDGF-BB.

#### 4.2.2. Fluorescence

**Figure 9.**
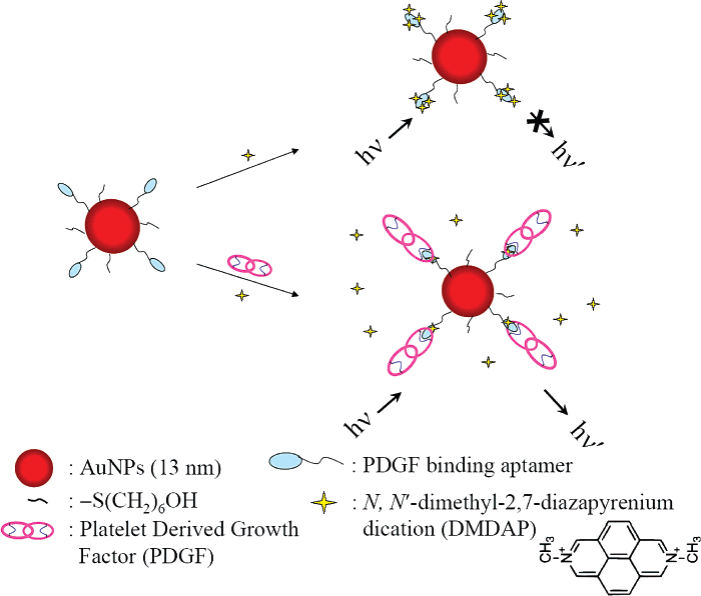
Schematic representations of PDGF nanosensors that operate based on modulation of the fluorescence resonance energy transfer between DMDAP and Apt-Au NPs. Reprinted with permission from Reference [127].

An Apt-Au NP based molecular light switching sensor was prepared for the analysis of PDGFs and their receptors in homogeneous solutions [127]. The PDGF binding aptamer has a unique structure with triple-helix conformation that allows *N,N*-dimethyl-2,7-diazapyrenium dication (DMDAP) and PDGF bindings (Figure 9).

The fluorescence of DMDAP was almost completely quenched by Apt-Au NPs when it intercalated with the aptamers. Owing to high magnitudes of increases (up to 40-fold) in the turn-on fluorescence signals of DMDAP/Apt-Au NP upon PDGFs binding, the approach was highly sensitive for the detection of PDGFs. The Apt-Au NPs also were effective selectors for enrichment of PDGF-AA from large-volume samples. The approach allowed detection of PDGF-AA at a concentration down to 8 pM. By conducting a competitive assay, determination of PDGF receptor-α with an LOD was to be 0.25 nM when using the DMDAP/Apt-Au NP as a probe.

A FRET-based sensor that Apt-QD (CdSe/ZnS) and black hole quencher (BHQ) acting separately as donor and acceptor was developed for detecting PDGF [128]. BHQ-bearing oligonucleotide (BHQ-oligonucleotide) molecules showing partial sequence matching to PDGF aptamer were attached to PDGF aptamers and PL quenching was obtained through FRET. By adding target PDGF-BB to the bioconjugates containing BHQs, PL recovery was detected due to detachment of BHQ-bearing oligonucleotide from the PDGF aptamer as a result of the difference in affinity to the PDGF aptamer. The detection limit of the sensor was ∼0.4 nM.

**Figure 10.**
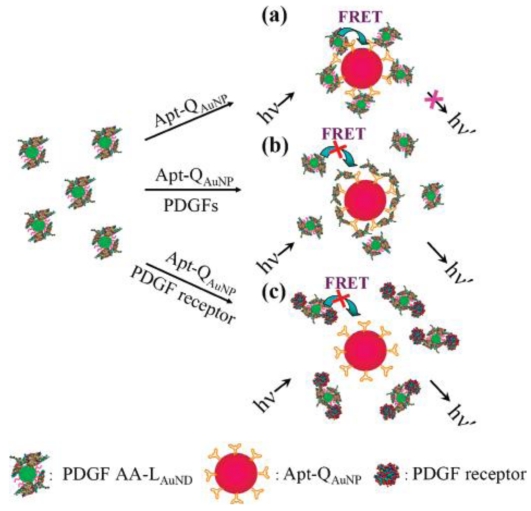
Schematic representations of PFGF and PDGF-receptor nanosensors that operate based on the modulation of the photoluminescence quenching between PDGF AA-Au NP and Apt-Au NP. h: Planck's constant; ν: frequency of light. Reprinted with permission from Reference [132].

In addition to strong SPR absorption properties, Au NPs having the dimensions smaller than 2.0-nm possess photoluminescence properties due to quantum confinement effects [64,65,129-133]. Preparation of water-soluble alkanethiol (RSH)-bound Au NPs (RS-Au NPs) having tunable photoluminescence wavelengths (501–618 nm), with quantum yields ranging from 0.0062 to 3.1% have been reported [64]. In addition, controlling the molar ratios of tetrakis(hydroxymethyl)-phosphonium chloride (THPC) to Au ions and of Ag ions to Au ions allows further preparation of different sizes of Au and Au/Ag NPs [133]. After they interacted with 11-mercaptoundecanoic acid (11-MUA), wavelength-tunable luminescent 11-MUA-Au NPs (500–640 nm) and 11-MUA-Au/Ag NPs (456–525 nm), respectively, were prepared [133]. The prepared luminescent and water-soluble 11-MUA-Au and -Au/Ag NPs offer several features for bioassays, including large Stokes-shifted and long luminescence lifetimes, sizes comparable to bipolymers, and good water solubility. Unlike semiconductive QDs, they are more compatible with biological systems and are not prepared from toxic precursors under vigorous conditions. In addition, bioconjugation of luminescent Au NPs are quite easy by taking advantage of strong Au-S bonding. Using two differently sized Au NPs, acting separately as donor and acceptor, homogeneous luminescence quenching assays were developed for the analysis of PDGF and its receptor [132]. Introduction of PDGF AA to a solution of 11-MUA-protected, 2.0-nm luminescent Au NPs leads to the preparation of PDGF AA-Au NP as the donor. Thiol-derivative PDGF binding DNA aptamers and 13-nm spherical Au NPs were used for preparation of the Apt-Au NP acceptor. In the assay, PDGF AA-Au NPs and Apt-Au NPs were mixed in the absence and presence of PDGF. Once luminescent PDGF AA-Au NPs and Apt-Au NPs were mixed, the two differently sized Au NPs become close, leading to occurrence of luminescence quenching through electron and/or energy transfer (Figure 10). As a result, the luminescence of PDGF AA-Au NPs at 520 nm decreased when luminescence quenching occurred between Apt-Au NP and PDGF AA-Au NP (Figure 10a). The PDGF AA-Au NP/Apt-Au NP-based molecular light switching system allowed analysis of PDGFs as well as PDGF α-receptor in separate homogeneous solutions (Figure 10b and 10c). In the presence of PDGFs, the interaction between Apt-Au NP and PDGF AA-Au NP decreased as a result of competitive reactions between the PDGFs and Apt-Au NP. Similarly, the interaction between Apt-Au NP and PDGF AA-Au NP reduced as a result of competitive reactions between PDGF α-receptor and PDGF AA-Au NP. The LODs for PDGF AA and PDGF α-receptor were 80 pM and 0.25 nM, respectively, resulting from a low background luminescence signal. When using the Apt-Au NP as selectors for (a) the enrichment of PDGF AA and (b) the removal of matrices possessing intense background fluorescence from cell media and urine samples, the LOD for PDGF AA decreased to 10 pM.

#### 4.2.3. Electrochemistry

Wang *et al.* reported an electrochemical detection approach for PDGF *via* sandwich structure and Au-NPs mediated amplification technique [134]. The sandwich structure was fabricated based on the fact that PDGF has two aptamer-binding sites, which makes it possible for one PDGF molecule to connect with two aptamers simultaneously. The electrochemical probes ([Ru(NH_3_)_5_Cl]^2+^) were employed for the signal readout *via* the electrostatic interaction between the positive probes ([Ru(NH_3_)_5_Cl]^2+^) and anionic phosphates of the Apt-Au NPs. It was found that this electrochemical system with sandwich structure and Au-NPs could significantly amplify the signal of electrochemical probe ([Ru(NH_3_)_5_Clrsqb;^2+^) for PDGF detection, and thus increased the detection sensitivity significantly. This PDGF detection approach obtained an extraordinarily low detection limit of 1 × 10^–14^ M for purified samples, and 1 × 10^–12^ M for contaminated-ridden samples or undiluted blood serum.

### 4.3. Other Proteins

A fluorescent aptasensor based on the magnetic separation for simultaneous detection thrombin and lyzome was proposed by Wang *et al.* [135]. Anti-thrombin aptamer and anti-lysozyme aptamer acting as the protein captor were individually immobilized onto magnetic NPs. The other antithrombin aptamer and anit-lysozyme aptamer that were separately labeled with rhodamine B and fluorescein, were employed as the protein reporters. By applying a sandwich detection strategy, the fluorescence responses at 515 nm and 578 nm were separately corresponding to lysozyme and thrombin with high selectivity and sensitivities. The LODs were 0.06 nM for thrombin and 0.2 nM of lysozyme, respectively.

A novel sandwich immunoassay that was designed to demonstrate the amplification effect of aptamer-Au NPs conjugates for detecting human immunoglobulin E (IgE) was reported by Wang *et al.* [136]. The amplification effect of the aptamer-Au NPs conjugates for human IgE was through SPR, with a LOD corresponding to 1 ng/mL. Another aptamer-based label free immunoassay for detecting IgE using CNT field effect transistor was developed by Maehashi *et al.* [137].

### 4.4. Cells

Aptamers selected by a cell-SELEX method can recognize their target molecules with high affinity and specificity. Aptamers have been selected for several cell types, including small cell and nonsmall cell lung cancer, liver cancer cells, and lymphocytic and myeloid leukemia cells [138-142]. When applying whole living cells as a target, a panel of aptamers can be generated simultaneously for the recognition of the unique molecular signatures of cancer cells through a cell-based SELEX process [142-148]. To overcome limitations in aptamer selection due to nonspecific binding of RNA or DNA pools to other sites on cancer cell surfaces than the desired target site, by using control cells to remove RNA or DNA sequences that do not bind specifically to cancer cells, it is possible to select molecular probes for cancer cells without knowing the detailed biochemical differences between cancer cells and healthycells [138]. These selected aptamers can then be used to distinguish cancer cells from normal cells and, moreover, to differentiate a particular tumor type from among various strains [142-148]. In addition, identified aptamers can then be employed as capture agents for cell separation and identification of their target proteins on the cell surface [138]. Therefore, aptamers generated from whole living cells are molecular probes for target cells on a molecular level. When bound with the membrane receptors of the cell lines, aptamers can be used for the discovery of biomarkers as disease signals. Because some of the selected aptamers possess weak binding affinity, and many disease cells have only low density target membrane proteins, especially in their early developmental stages, the approaches do not provide sensitively detect cancer cells by aptamers. In order to increase signal strength and enhance binding affinity, multivalent ligand scaffolded Apt-Au NPs with ultrasensitive optical detection have been developed for rapid, sensitive and economical cell recognition [145].

Huang *et al.* demonstrated the potential use of Apt-Au NPs for cancer cell detection [149]. The Au NPs acted as contrast agents using a simple and inexpensive conventional dark-field optical microscope (DFM). The aptamers were used as recognition units that had a high affinity toward PDGF. The expression of PDGF in normal cells occurs at undetectable or low levels, whilst in cells with malignancies and developmental abnormalities, it is over-expressed. Through specific binding of the aptamers toward PDGF, aggregation of the Apt-Au NPs in the cytoplasm of MDA-MB-231 and Hs578T cells (cancer cells) led to the generation of a greater intense scattered light than H184B5F5/M10 cells (normal cells) upon photo-illumination. In addition, the presence of Apt-Au NPs suppressed the proliferation of MDA-MB-231 cancer cells, but not H184B5F5/M10 cells. Dye doped silica NPs possess some key advantages over conventional organic dyes, such as much stronger optical signal than a single-dye molecule, and more stable against photobleaching [150,151]. Moreover, the surface of silica NPs provides a robust and stable shell, usually with a versatile composition that allows easy manipulation and feasible functionalization, either through physical adsorption or covalent attachment [150,151]. Fluorescent silica NPs conjugated with aptamers were used in the detection of cancer cells, showing their potential in tumor diagnosis [152,153]. Recently, Chen *et al.* used SiO_2_ NPs conjugated with aptamers for multiplexed monitoring of cancer cells through fluorescence resonance energy transfer (FRET) [154]. By changing the doping ratio of three different dyes (FAM, R6G and ROX), the FRET-mediated emission signatures could be tuned such that the NPs would exhibit multiple colors upon excitation with a single wavelength. The NPs were modified with aptamers that are specific for different cancer cell line such as T-cell leukemia and B-cell lymphoma. Zheng *et al.* demonstrated composite nanomaterials, termed an aptamer nano-flare, for direct quantification of an intracellular analyte in living cells [155]. These nanostructures consisted of Au NPs that were functionalized with a dense monolayer (∼8.4 pmol/cm^2^) of chemisorbed aptamer oligonucleotides hybridized to fluorophore (Cy5) labeled flares. The flare oligonucleotide was designed to bind to the aptamer that was attached to the surface of the Au NP. In this bound state, the fluorescence of the flare strand was quenched by the Au NPs. In the presence of the ATP target molecules, ATP bound to the aptamer causing a conformational change and resulting in a new folded secondary structure. This folded structure disrupted the Watson-Crick base-pairing between the aptamer and the flare, which caused flares to be liberated with an increase in fluorescence due to the greater distance of the flare from the gold surface. Aptamer nanoflares were sensitive to physiologically relevant changes in ATP concentrations (0.1–3 mM) and showed a high selectivity for ATP when compared to other nucleoside-triphosphate analogs. Aptamer nano-flares readily entered HeLa cells where they could be used to directly quantify intracellular ATP levels, showing their potential for cell imaging and sorting studies.

## 5. Conclusions

Aptamer-based nanosensors have already been successfully employed for highly sensitive and selective detection of molecules through various transducing approaches such as absorption, fluorescence, mass spectrometry, electrochemistry, and SERS. The Apt-NMs based sensor systems successfully detect a number of analytes of interest, including small organic molecules, metal ions, and proteins, by taking advantages of highly selective interactions of the aptamer with the target analytes and high amplification of signals by the unique optical, electrical, and magnetic properties of various NMs. In addition, the multivalent effect due to the ultrahigh densities of aptamer molecules on the local surface of the NMs enhance the binding constant more than two orders, which leads to improves in the sensitivity. Successful examples presented in this review clearly show that Apt-NMs are effective materials for fabrication of new sensing devices for diagnostics of biological samples and monitoring of environmental samples.

It has been known that the structure and specificity of aptamers are often dependent on pH, ionic strength, and viscosity. Critical conditions that may not be compatible to biological and environmental conditions are usually required when Apt-NMs are used. In addition, nonspecific interactions of Apt-NMs with the sample matrices may occur. As a result, their applications may be limited. To make ideal Apt-NMs, fine control of their sizes, shapes, ligand densities, and surface species (stabilizers) is necessary. Use of multiple molecules (aptamers, small molecules, polymers, and biopolymers) to prepare functionalized NMs should be worth trying to overcome nonspecific interactions and to stabilize the functionalized NMs, while retaining their biological function. In order to improve sensitivity, NMs possessing greater molar absorption coefficient and greater quantum yields in the near-infrared region are beneficial when developing optical sensors. Several NMs such as type-II QDs that fluoresce in the NIR region are good candidates for developing sensors. While anisotropic NPs such as nanorods, nanowires and cubes are useful for developing SERS based sensors. Metallic nanowires and nanotubes are possible for developing electrochemistry based sensors. With advances in nanosciences and life sciences, one can foresee significant progress in developing novel sensor systems and their applications.

## Figures and Tables

**Table 1.** Representative aptamers used for the detection of small analytes.

**Probe**	**Target**	**Detection**	**Time**	**LOD**	**Ref**
Apt-Au NPs	adenosine	colorimetric	10 s	0.1 mM	[32]
Apt-Au NPs	adenosine	colorimetric	5 min	20 μM	[33]
Apt-Au NPs	adenosine	colorimetric	1 min	10 μM	[34]
Apt-Au NPs	adenosine	colorimetric	10 min	20 μM	[35]
Apt-Au NPs	adenosine	SPR	30 min	1 nM	[36]
Apt-Au NPs	adenosine	electrochemical	90 min	180 pM	[37]
Apt-QD & Au NPs	adenosine	fluorescence	1 min	50 μM	[38]
Apt-Au NPs	cocaine	colorimetric	10 min	20 μM	[39]
Apt-Au NPs	cocaine	colorimetric	10 s	25 μM	[32]
Apt-Au NPs	cocaine	colorimetric	5 min	10 μM	[33]
Apt-QD	cocaine	fluorescence	1 min	120 μM	[38]
Apt-QD & Atto 590	cocaine	fluorescence	15 min	1 μM	[40]
Apt-QD & Cy5 & Iowa Black RQ	cocaine	fluorescence	-	0.5 μM	[41]
Au NPs & Fc-Apt	cocaine	electrochemical	5 min	0.5 μM	[42]
Apt-Au NPs	ATP	colorimetric	30 min	0.6 μM	[43]
Apt-Au NPs	ATP	colorimetric	30 min	10 nM	[44]
Apt-Au NPs & Au NPs	ATP	SALDI-MS	10 min	0.48 μM	[45]
Apt-QD & Cy5	ATP	fluorescence	-	24 μM	[46]
Apt-SiO_2_@Fe_3_O_4_	AMP	fluorescence	-	0.1 μM	[47]
Apt-Au NPs & DNA-Au NP-Hg^2+^ aggregates	cysteine	colorimetric	-	100 nM	[48]
Apt-Au NPs	Hg^2+^	colorimetric	-	100 nM	[49]
Apt-Au NPs	Hg^2+^	colorimetric	30 min	10 nM	[50]
Apt-Au NPs	Hg^2+^	colorimetric	10 min	25 nM	[51]
Apt-Au NPs	Hg^2+^	colorimetric	5 min	0.6 nM	[31]
Apt-Au NPs	Hg^2+^	colorimetric	-	1 μM	[52]
Apt-Au NP	Hg^2+^	colorimetric	-	250 nM	[53]
Apt-Au NPs & DNAzyme	Hg^2+^	colorimetric	-	1 nM	[54]
Au NPs & Dye-Apt	Hg^2+^	fluorescence	30 min	40 nM	[55]
Apt-Au NPs	K^+^	colorimetric	4 min	1 mM	[56]
Apt-Au NPs	K^+^	colorimetric	10 min	0.5 mM	[35]
Apt-Au NPs & DNAzyme	Pb^2+^	colorimetric	-	100 nM	[57]
Apt-Au NPs & DNAzyme	Pb^2+^	colorimetric	10 min	0.4 μM	[58]
Apt-Au NPs & DNAzyme	Pb^2+^	colorimetric	5 min	0.1 μM	[59]
Apt-Au NPs & DNAzyme	Pb^2+^	colorimetric	6 min	120 nM	[60]
